# Polymorphisms in the vascular endothelial growth factor gene and the risk of diabetic retinopathy in Chinese patients with type 2 diabetes

**Published:** 2011-11-24

**Authors:** Xiufen Yang, Yu Deng, Hong Gu, Apiradee Lim, Ariunzaya Altankhuyag, Wei Jia, Kai Ma, Jun Xu, Yanhong Zou, Torkel Snellingen, Xipu Liu, Ningli Wang, Ningpu Liu

**Affiliations:** 1 Beijing Tongren Eye Center, Beijing Tongren Hospital, Capital Medical University, Beijing Ophthalmology and Visual Sciences Key Laboratory, Beijing, China; 2 Department of Mathematics and Computer Science, Faculty of Science and Technology, Prince of Songkla University, Pattani Campus, Pattani, Thailand; 3 Sekwa Eye Hospital, Beijing, China; 4 Department of Ophthalmology, First Hospital of Tsinghua University, Beijing, China

## Abstract

**Purpose:**

To investigate whether single nucleotide polymorphisms (SNPs) in the vascular endothelial growth factor (*VEGF*) gene are associated with diabetic retinopathy (DR) in a cohort of Chinese patients with type 2 diabetes mellitus (T2DM).

**Methods:**

A total of 268 patients with T2DM (129 with DR and 139 without DR) were recruited and enrolled in the study. Patients with T2DM were assigned to a DR group or a diabetic-without-retinopathy group, based on the duration of diabetes and grading of fundus images. Genotypes of eight SNPs in the *VEGF* gene (rs699947, rs833061, rs13207351, rs2010963, rs833069, rs2146323, rs3025021, and rs3025039) were analyzed using a mass-array genotyping system, and an association study was performed.

**Results:**

After adjusting for covariates, a significant association of DR was observed with the homozygous genotype of the minor allele for promoter SNPs rs699947 (odds ratio (OR)=3.54, 95% confidence interval (CI): 1.12–11.19), rs833061 (OR=3.72, 95% CI: 1.17–11.85) and rs13207351 (OR=3.76, 95% CI: 1.21–11.71). A significant association of DR was also observed with haplotype ACA, as defined by minor alleles of promoter SNPs rs699947, rs833061, and rs13207351 (OR=1.52, 95% CI: 1.03–2.24), and haplotype GAA, as defined by SNPs rs2010963, rs833069, and rs2146323 (OR=1.62, 95% CI: 1.08–2.41).

**Conclusions:**

Our data suggest that polymorphisms in the promoter region of the *VEGF* gene increase the risk of DR in Chinese patients with T2DM.

## Introduction

Diabetic retinopathy (DR) accounts for 8% of legal blindness in the United States, making it the leading cause of legal blindness in working-age adults [1]. The accumulated evidence suggests that the severity and progression of DR are influenced by genetic and environmental factors. Vascular endothelial growth factor (VEGF), an endothelial cell-specific mitogen, has been implicated as a major contributor to the development of DR [2,3]. Both hypoxia and hyperglycemia are potent stimuli for VEGF protein expression [4-6], and the vitreous concentration of VEGF has been shown to be higher in patients with DR [7-10]. Elevated VEGF and its receptor expression have also been demonstrated in diabetic retinas [11]. Moreover, VEGF antagonists are able to reduce retinal vascular permeability and neovascularization, thus inhibiting the development of DR [12,13].

The human *VEGF* gene, located on chromosome 6, is highly polymorphic, with seven introns and eight exons. Single nucleotide polymorphisms (SNPs) in the promoter and the 5′ untranslated region (UTR) of the *VEGF* gene have been shown to influence the expression of the VEGF protein [14-22]. Several longitudinal [23,24] and case-control [14,18,22,25-32] studies also suggest that SNPs in VEGF gene mediate genetic predisposition to DR. The results from different studies, however, are conflicting and inconclusive [14,23-34]. In the present study, we aimed to investigate whether common polymorphisms in the *VEGF* gene are associated with DR in a cohort of Chinese patients with type 2 diabetic mellitus (T2DM).

## Methods

### Study participants and clinical evaluation

Patients with T2DM were recruited between November 2009 and September 2010 from the Desheng community of urban Beijing. The study protocol was approved by the Ethics Committee of Beijing Tongren Hospital and adhered to the tenets of the Declaration of Helsinki. Written informed consent was obtained from all participants before their enrollment. Diabetes was defined as a self-reported history of physician-diagnosed T2DM treated with insulin, oral hypoglycemic agents, or diet only, or by a fasting plasma glucose (FPG) concentration of 7.0 mmol/l (126 mg/dl) or more in at least two previous examinations or a random plasma glucose concentration of ≥11.1 mmol/l (200 mg/dl). All subjects underwent a standardized evaluation consisting of a questionnaire, ocular and anthropometric examinations, and a laboratory test. The questionnaire elicited basic information (age, sex, ethnicity, income, education), lifestyle information (such as smoking and alcohol intake), health status information (such as the use of insulin therapy and any history of systemic diseases), and family history of diseases. Anthropometric parameters included bodyweight and height, waist and hip circumference, and three measurements, 5 min apart, of blood pressure in a resting state. Body mass index (BMI, kg/m^2^) [35] and waist-to-hip ratio were calculated. A comprehensive ophthalmological examination included corrected visual acuity, slit-lamp biomicroscopy, and fundus photography [36,37]. Seven fields 30° color fundus photographs with stereoscopic images of the optic disc and macula were taken through the dilated pupils of each patient using a digital fundus camera (Zeiss Visucam Pro, Oberkochen, Germany).

Based on the duration of diabetes and grading of fundus photographs, patients were assigned to the diabetic-without-retinopathy (DWR) group if they had more than 10 years’ duration of T2DM with no signs of DR (microaneurysms, hemorrhages, exudates) or if they had more than 15 years’ duration of T2DM with fewer than five microaneurysms [29,38]. Patients with five or more microaneurysms in at least one eye were assigned to the DR group. Patients who did not meet the DWR or DR criteria were excluded from this study. The duration of diabetes was defined as the interval between the first diagnosis of diabetes and the time of enrollment in the present study.

### Laboratory assays

Fasting blood samples were collected for measurement of FPG, glycosylated hemoglobin A1c (HbA1c), creatinine, uric acid, and lipid profile (levels of total cholesterol, triglycerides, and high-density and low-density lipoprotein cholesterol), which were measured in an automated system with reagents for routine biomarkers. HbA1c was assessed by the enzymatic method using a Hitachi analyzer 7080 (Ibaraki, Japan). A first-void, midstream morning spot urine sample was collected, and albuminuria was measured by immunonephelometry with a Roche/Cobas C501 analyzer (Ibaraki, Japan). High albuminuria was defined as ≥20 mg/l [39].

### Genotyping for vascular endothelial growth factor polymorphisms

Blood samples were collected from all participants and stored at −80 °C before DNA extraction. Genomic DNA was isolated from venous blood leukocytes using a genomic DNA extraction and purification kit (TIANamp Swab DNA Kit; Tiangen Biotech, Beijing, China). Eight SNPs in the *VEGF* gene (NCBI Reference Sequence: NG008732.1) were genotyped, including rs699947 (−2578C>A) at the promoter region, rs833061 (−1498T>C) at the promoter region, rs13207351 (−1190G>A) at the promoter region, rs2010963 (−634C>G) at 5′ UTR, rs833069 (3596A>G) at intron 2, rs2146323 (6112C>A) at intron 2, rs3025021 (10180T>C) at intron 6, and rs3025039 (13553C>T) at 3′ UTR. The nucleotide sequence variation in all SNPs was numbered from the start of translation. Study participants were genotyped for the SNPs using Sequenom MassARRAY technology (Bioyong Technologies, Beijing, China).

### Statistics

Statistical analysis was performed using the R statistical analysis package. Baseline characteristics of diabetic patients in the DR and DWR groups were compared using a *t*-test for continuous variables or a chi-square test for categorical variables. The genotype frequencies of *VEGF* were checked for the Hardy–Weinberg equilibrium using a chi-square test. The chi-square test was also used to analyze the distribution of genotypes and alleles. When the expected frequency was less than 5, Fisher’s exact test was used. The pairwise linkage disequilibrium was calculated using Haploview, version 4.2 (Broad Institute, Cambridge, MA) [40]. Univariate and multivariate logistic regression analyses were performed to identify the association between *VEGF* SNPs/haplotypes and the presence of DR. Results were expressed as p values, odds ratios (OR), and 95% confidence intervals (CI). Statistical significance was set at p<0.05.

## Results

A total of 268 patients (129 in the DR group and 139 in the DWR group) were selected for the present study. Clinical features of the study subjects are presented in Table 1. Compared with the DWR group, those with DR had significantly higher levels of creatinine (p=0.02), FPG (p=0.003), and HbA1c (p<0.001). Patients in the DR group had higher percentages of high albuminuria (p<0.001) and were more likely to use insulin (p<0.001) than DWR patients. There was no statistically significant difference between the DR and DWR groups in terms of gender, duration of diabetes, age of diabetic onset, BMI, waist-to-hip ratio, blood pressure, serum levels of uric acid, or lipid profile.

**Table 1:** Clinical and biochemical markers for the studied groups.

**Clinical characteristics**	**DWR (n=139)**	**DR (n=129)**	**p value**
Age of diabetic onset (years)	50.45±8.00	48.68±9.58	0.1
Sex (Male/Female)	58/81	57/72	0.78
Duration of diabetes (years)	15.06±4.38	14.59±7.52	0.52
BMI (kg/m2)	24.55±4.00	24.79±4.36	0.63
WHR	0.92±0.06	0.93±0.07	0.37
High albuminuria(-/+)	121/17	81/48	< 0.001
Systolic blood pressure (mmHg)	136±16.97	137.1±16.15	0.59
Diastolic blood pressure (mmHg)	77.38±8.82	78.92±10.05	0.33
Insulin therapy (yes/no)	47/92	81/48	< 0.001
HbA1c (%)	7.07±1.39	7.93±1.67	< 0.001
FPG (mmol/l)	8.23±2.46	9.23±2.97	0.003
Creatinine (µmol/l)	65.14±16.49	71.49±24.42	0.02
Uric acid (µmol/l)	275.8±77.83	277.7±68.38	0.84
Cholesterol (mmol/l)	5.22±0.95	5.26±1.13	0.7
Triglycerides (mmol/l)	1.67±1.09	1.67±1.17	0.75
HDL cholesterol (mmol/l)	1.21±0.29	1.23±0.32	0.65
LDL cholesterol (mmol/l)	3.08±0.79	3.08±0.86	0.98

The data are expressed as mean±standard deviation (SD) or number of subjects. Differences between diabetic retinopathy (DR) and diabetic without retinopathy (DWR) groups were compared using *t*-test or χ^2^ test. BMI, body mass index; WHR, waist and hip ratio; HbA1c, glycosylated hemoglobin A1c; FPG, fasting plasma glucose; HDL, high-density lipoprotein; LDL, low-density lipoprotein.

Table 2 presents a comparison of genotype and allele frequencies between the DWR and DR groups. More than 98% of the subjects were successfully genotyped for each SNP using mass spectrometry. The genotype distribution of rs3025021 was in Hardy–Weinberg equilibrium in the DWR group (p=0.28) but showed a slight deviation from it in the DR group (p=0.031). The genotype distribution for all the other seven SNPs tested was in Hardy–Weinberg equilibrium in both the DR and DWR groups (p≥0.069). A strong association with DR was detected for SNPs rs699947, rs833061, and rs13207351 at the promoter region of the *VEGF* gene. Compared to the DWR group, the DR group had significantly higher frequencies for the A allele of rs699947 (p=0.020), the C allele of rs833061 (p=0.032), and the A allele of rs13207351 (p=0.029). The genotypic distribution between the DR and DWR groups was statistically significantly different for rs699947 (p=0.022), rs833061 (p=0.021), and rs13207351 (p=0.017). Moreover, a statistically significant difference between the DR and DWR groups was observed in the allele frequency for intronic SNPs rs2146323 (p=0.021) and rs3025021 (p=0.021); however, the distribution of genotypes was only marginally significant for rs2146323 (p=0.09) and rs3025021 (p=0.067). No statistically significant difference was found for the allele frequencies or genotypic distributions in SNPs rs833069, rs3025039, and rs2010963.

**Table 2:** Allele and genotype frequencies of vascular endothelial growth factor (*VEGF*) polymorphisms in the studied groups.

**SNP**	**Location of SNPs**	**Sample size**	**Allele and genotype**	**DR**	**DWR**	**p value**
rs699947	Promoter	267	A	80 (31.0)	61 (22.1)	0.020
			CC	66 (51.2)	82 (59.4)	0.022
			CA	47 (36.4)	51 (37.0)	
			AA	16 (12.4)	5 (3.6)	
				H-W p=0.10	H-W p=0.47	
rs833061	Promoter	265	C	78 (30.7)	62 (22.5)	0.032
			TT	65 (51.2)	81 (58.7)	0.021
			CT	46 (36.2)	52 (37.7)	
			CC	16 (12.6)	5 (3.6)	
				H-W p=0.10	H-W p=0.47	
rs13207351	Promoter	264	A	80 (31.0)	61 (22.6)	0.029
			GG	66 (51.2)	79 (58.5)	0.017
			GA	46 (35.7)	51 (37.8)	
			AA	17 (13.2)	5 (3.7)	
				H-W p=0.069	H-W p=0.47	
rs2010963	5′ UTR	266	C	112 (43.4)	124 (45.3)	0.67
			GG	36 (27.9)	39 (28.5)	0.61
			GC	74 (57.4)	72 (52.6)	
			CC	19 (14.7)	26 (19)	
				H-W p=0.073	H-W p=0.50	
rs833069	Intron 2	268	G	117 (45.3)	130 (46.8)	0.74
			GG	23 (17.8)	29 (20.9)	0.80
			GA	71 (55)	72 (51.8)	
			AA	35 (27.1)	38 (27.3)	
				H-W p=0.22	H-W p=0.74	
rs2146323	Intron 2	266	A	76 (29.7)	58 (21.0)	0.021
			CC	67 (52.3)	88 (63.8)	0.09
			CA	46 (35.9)	42 (30.4)	
			AA	15 (11.7)	8 (5.8)	
				H-W p=0.14	H-W p=0.44	
rs3025021	Intron 6	264	C	225 (87.9)	219 (80.5)	0.021
			TT	4 (3.1)	7 (5.1)	0.067
			CT	23 (18)	39 (28.7)	
			CC	101 (78.9)	90 (66.2)	
				H-W p=0.031	H-W p=0.28	
rs3025039	3′ UTR	268	T	50 (19.4)	53 (19.1)	0.93
			CC	83 (64.3)	89 (64.0)	0.88
			CT	42 (32.6)	47 (33.8)	
			TT	3 (3.1)	3 (2.2)	
				H-W p=0.79	H-W p=0.29	

Data are expressed as number (%). Genotype distributions for SNPs were in Hardy–Weinberg (H-W) equilibrium, except rs3025021 in diabetic retinopathy (DR) group. The p value represents comparison between DR and diabetic without retinopathy (DWR) groups, using χ^2^ or Fisher’s exact test. UTR indicates untranslated region.

Table 3 presents data from the logistic regression analysis. After adjusting for the age of diabetic onset, high albuminuria, insulin use, HbA1c, FPG, and creatinine levels, the association with DR remained statistically significant for the homozygous genotype of the minor alleles at the promoter SNPs rs699947 (OR=3.54; 95% CI: 1.12–11.19), rs833061 (OR=3.72; 95% CI: 1.17–11.85), and rs13207351 (OR=3.76; 95% CI: 1.21–11.71). No statistically significant association with DR was observed after the adjustment for the homozygous genotype of minor alleles at the intronic SNPs rs3025021 (OR=2.25; 95% CI: 0.81–6.29) or rs2146323 (OR=0.93; 95% CI: 0.23–3.82).

**Table 3:** Association between vascular endothelial growth factor (*VEGF*) polymorphisms and risk of diabetic retinopathy in patients with type 2 diabetes mellitus.

**SNP**	**Location of SNPs**	**Genotype**	**Crude OR (95% CI)**	**Adjusted OR (95% CI)**	**p (Wald's test)**
rs699947	Promoter	CC	1	1	
		CA	1.14 (0.69, 1.91)	1.04 (0.59, 1.85)	0.89
		AA	3.98 (1.38, 11.42)	3.54 (1.12, 11.19)	0.031
rs833061	Promoter	TT	1	1	
		CT	1.10(0.66, 1.84)	1.00 (0.56, 1.79)	0.99
		CC	3.99 (1.39, 11.46)	3.72 (1.17, 11.85)	0.026
rs13207351	Promoter	GG	1	1	
		AG	1.08 (0.64, 1.81)	0.98 (0.55, 1.74)	0.95
		AA	4.07 (1.43, 11.62)	3.76 (1.21, 11.71)	0.02
rs2146323	Intron 2	CC	1	1	
		CA	1.46 (0.86, 2.47)	1.31 (0.73, 2.35)	0.37
		AA	2.43 (0.97, 6.08)	2.25 (0.81, 6.29)	0.12
rs3025021	Intron 6	TT	1	1	
		CT	0.54 (0.30, 0.97)	0.50 (0.11, 2.27)	0.37
		CC	0.59 (0.16, 2.17)	1.08 (0.26, 4.44)	0.92

Multiple logistic regression analysis was used to calculate odds ratio (OR) and 95% confidence interval (CI) by comparing diabetic retinopathy (DR) group with diabetic without retinopathy (DWR) group. Adjusted OR represents data after adjustment for covariates including the age of diabetic onset, high albuminuria, insulin use, glycosylated hemoglobin A1c, fasting plasma glucose, and creatinine.

**Figure 1:**
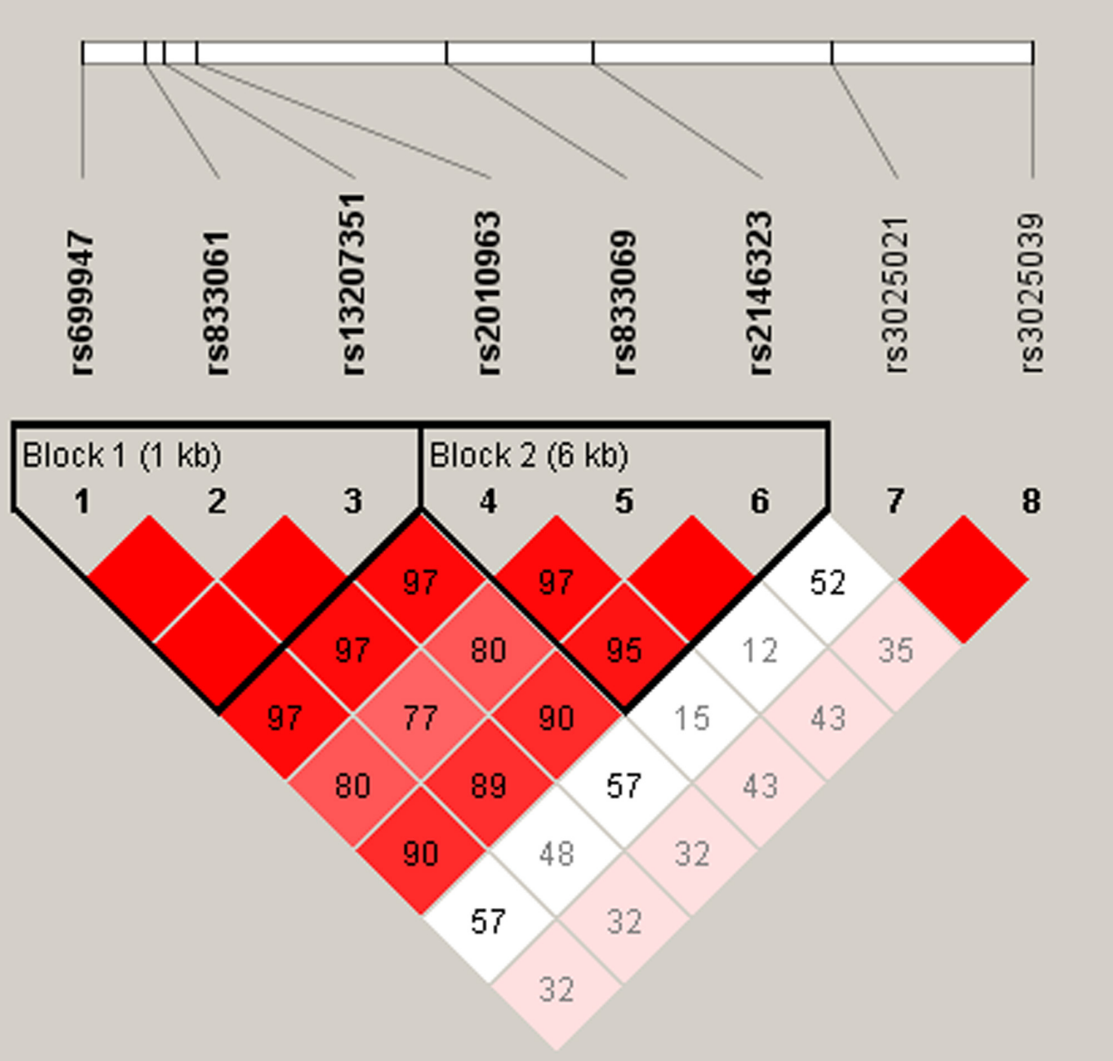
Linkage disequilibrium plot generated by Haploview 4.2 software. Two haplotype blocks (bold) were identified for single nucleotide polymorphisms (SNPs) in the vascular endothelial growth factor (*VEGF*) gene. Linkage disequilibrium (LD) is displayed as pairwise D’ values multiplied by 100 and given for each SNP combination [40]. Shading represents the magnitude and significance of pairwise LD, with a red-to-white gradient reflecting higher-to-lower LD values. Red diamond without a number corresponds to D’ values of 1.0.

The linkage disequilibrium analysis revealed two SNP blocks (Figure 1). Block 1 included three promoter SNPs: rs699947, rs833061, and rs13207351. Block 2 included the SNPs rs2010963 at 5′ UTR, rs833069 at intron 2, and rs2146323 at intron 2. Five major haplotypes with frequencies of more than 0.03 were estimated, and each was compared between the DR and DWR groups (Table 4). In block 1, the frequency of haplotype ACA, defined by the minor alleles of the promoter SNPs rs699947, rs833061, and rs13207351, was 30.7% in the DR group and 22.6% in the DWR group (p=0.036, OR=1.52, 95% CI: 1.03–2.24). In block 2, the frequency of haplotype GAA, defined by the minor alleles of the SNPs rs2010963, rs833069, and rs2146323, was significantly higher in the DR group than in the DWR group (p=0.018, OR=1.62, 95% CI: 1.08–2.41).

**Table 4:** Haplotype association analysis between vascular endothelial growth factor (*VEGF*) polymorphisms and risk of diabetic retinopathy in patients with type 2 diabetes mellitus.

**Haplotype blocks**	**DR group (%)**	**DWR group (%)**	**OR (95%CI)**	**χ^2^**	**p value**
**Block 1**					
CTG	69.3	77.4	0.66 (0.45, 0.97)	4.42	0.036
ACA	30.31	22.6	1.52 (1.03, 2.24)	4.42	0.036
**Block 2**					
CGC	42.9	44.9	0.95 (0.67, 1.34)	0.1	0.76
GAC	24.2	32.1	0.69 (0.47, 1.01)	3.73	0.054
GAA	29.3	20.8	1.62 (1.08, 2.41)	5.59	0.018

Data were given as frequency of each haplotype within diabetic retinopathy (DR) group or diabetic without retinopathy (DWR) group. Block 1 included promoter SNPs rs699947, rs833061, and rs13207351, whereas block 2 included SNPs rs2010963 at 5′ UTR, rs833069 at intron 2, and rs2146323 at intron 2. Only haplotypes with frequency over 3% were included in this table. OR indicates odds ratio and CI refers to confidence interval.

## Discussion

In the present study, we investigated the possible association between *VEGF* polymorphisms and the presence of DR in a well defined cohort of Chinese patients with T2DM. Eight SNPs, previously shown to be associated with altered VEGF protein production or risk of DR [14-22], were selected for the study. Our data support the observation that polymorphisms in the promoter region of the *VEGF* gene are associated with the risk of DR in diabetic patients.

As previously noted, the association between VEGF polymorphisms and the presence or severity of DR is inconclusive and conflicting either in the same or different populations [14,18,22-34]. As shown in Table 5, for example, rs833061 at the promoter region had been shown to be associated with the risk of DR in an Indian study [29], but such an association was not reported among Japanese [14] or Caucasians [22]. Another promoter polymorphism rs13207351 was found to be associated with DR in Caucasians [27] but not in Indian or Japanese populations [14,29]. Additionally, rs699947 at the promoter region was associated with DR in Koreans [32] and marginally in Japanese [26,31], but the data were inconclusive for Caucasians [23,25,33]. The disparities among the various studies may be due to sampling bias, differences in the inclusion criteria of cases, demographic factors, or differences in ethnic backgrounds [41,42]. In the present study of Chinese patients with T2DM, the three SNPs tested—rs699947, rs833061, and rs13207351—at the *VEGF* promoter region showed significant associations with DR susceptibility. Compared to the wild-type genotypes, the homozygous genotype of the minor alleles at rs699947, rs833061, and rs13207351 conferred, respectively, 3.54-, 3.72-, and 3.76-fold increases in the risk of DR in the study cohort. These same three SNPs showed strong linkage disequilibrium. Haplotypes containing minor alleles of these three promoter SNPs (A for rs699947, C for rs833061, and A for rs13207351) were also significantly associated with the risk of DR in the present study, suggesting that the association might lie within the linkage disequilibrium bin rather than with the individual SNPs [25,43].

**Table 5:** Genotype frequencies of the common promoter polymorphisms in the vascular endothelial growth factor (*VEGF*) gene in various published studies.

**SNP**	**Reference**	**Study population**	**Type of diabetes**	**Sample size (DR/DWR)**	**DR (%)**			**DWR (%)**			**p value**
rs833061					TT	TC	CC	TT	TC	CC	
	[14]	Japanese	2	150/118	52.7	38.7	8.7	44.1	48.3	7.6	NS
	[29]	Indian	2	120/90	30	67.5	2.5	67.8	32.2	0	0.0001
	[22]	Polish	2	154/61	3.2	55.6	41.2	4.9	55.7	39.3	NS
	Present study	Chinese	2	127/138	51.2	36.2	12.6	58.7	37.7	3.6	0.021
rs13207351					GG	GA	AA	GG	GA	AA	
	[14]	Japanese	2	150/118	52.7	38.7	8.7	44.1	48.3	7.6	NS
	[29]	Indian	2	120/90	55	25.8	19.2	61.1	25.6	13.3	0.5
	[27]	British	1 & 2	45/61	18	47.5	34.4	20	15.6	64.4	0.002
	Present study	Chinese	2	129/135	51.2	35.7	13.2	58.5	37.8	3.7	0.017
rs699947					CC	CA	AA	CC	CA	AA	
	[31]	Japanese	2	175/203	54.3	40	5.7	45.8	44.8	9.4	0.088
	[26]	Japanese	2	177/292	48	39.5	12.5	55.9	36.6	7.5	0.12
	[32]	Korean	2	253/134	48.6	45.5	5.9	68.7	26.9	4.4	<0.001
	[33]	Finland	1 & 2	131/98	19	48	33	32	40	28	0.17
	[25]	Australian	1	76/94	23	47	31	28	46	26	0.49
			2	139/187	23	54	23	25	50	25	0.14
	Present study	Chinese	2	129/138	51.2	36.4	12.4	59.4	37	3.6	0.022

DR, diabetic retinopathy; DWR, diabetic without retinopathy (DWR). NS indicates not significant.

Other SNPs at the intron region, where there can be intronic enhancers or silencers that regulate VEGF transcription [27], have not been extensively investigated [23,24,27]. In the present study, the mutant alleles of two intronic SNPs rs2146323 and rs3025021 showed significant association with DR. However, difference in genotype distribution of these two intronic SNPs between DR and DWR groups was only marginally significant in the study cohort. Since previous studies on these two intronic SNPs also generated contradicting results in Caucasians [23,27] and Japanese [24], their role in the pathogenesis of DR would need to be further explored. We did not find associations between DR susceptibility and the other three tested SNPs (rs833069, rs3025039, and rs2010963) in the present study.

The mechanism through which SNPs affect DR susceptibility is not known. However, promoter SNPs rs699947 and rs833061 have been reported to alter the VEGF promoter activity or affect levels of mRNA expression [15,17,19-21]. Patients carrying the risk C allele of *VEGF* polymorphism rs833061 have been shown to exhibit increased VEGF promoter activity [15,19]. The functional role of rs699947 is reported contradictive among studies, with patients carrying the C allele genotype showing both low [20] and high [17,21] VEGF levels. One of the possible explanations for the conflicting results of functional studies may lie in the fact that it is not an individual SNP but a combined haplotype that is more important influencing the gene function and DR susceptibility. Haplotypes might be more predictive and informative than individual SNPs [44].

In summary, our data suggest that the SNPs rs699947, rs833061, and rs13207351 at the promoter region of the *VEGF* gene might predispose Chinese patients with T2DM to the risk of DR. However, this is a small sample study, and further studies with a larger sample size are needed to confirm this observation. Moreover, functional studies are needed to understand the mechanisms by which *VEGF* polymorphisms alter susceptibility to DR.
